# Non-Integrating Lentiviral Vectors in Clinical Applications: A Glance Through

**DOI:** 10.3390/biomedicines10010107

**Published:** 2022-01-05

**Authors:** Narmatha Gurumoorthy, Fazlina Nordin, Gee Jun Tye, Wan Safwani Wan Kamarul Zaman, Min Hwei Ng

**Affiliations:** 1 Centre for Tissue Engineering and Regenerative Medicine (CTERM), Universiti Kebangsaan Malaysia Medical Centre (UKMMC), 56000 Kuala Lumpur, Malaysia; nmatha27@gmail.com (N.G.); angela@ukm.edu.my (M.H.N.); 2 Institute for Research in Molecular Medicine (INFORMM), Universiti Sains Malaysia (USM), 11800 Gelugor, Malaysia; geejun@usm.my; 3 Department of Biomedical Engineering, Faculty of Engineering, Universiti Malaya, 50603 Kuala Lumpur, Malaysia; wansafwani@um.edu.my

**Keywords:** HIV, integrate-deficient, lentiviral-vector, clinical application, vaccinations, NILV

## Abstract

Lentiviral vectors (LVs) play an important role in gene therapy and have proven successful in clinical trials. LVs are capable of integrating specific genetic materials into the target cells and allow for long-term expression of the cDNA of interest. The use of non-integrating LVs (NILVs) reduces insertional mutagenesis and the risk of malignant cell transformation over integrating lentiviral vectors. NILVs enable transient expression or sustained episomal expression, especially in non-dividing cells. Important modifications have been made to the basic human immunodeficiency virus (HIV) structures to improve the safety and efficacy of LVs. NILV-aided transient expression has led to more pre-clinical studies on primary immunodeficiencies, cytotoxic cancer therapies, and hemoglobinopathies. Recently, the third generation of self-inactivating LVs was applied in clinical trials for recombinant protein production, vaccines, gene therapy, cell imaging, and induced pluripotent stem cell (iPSC) generation. This review discusses the basic lentiviral biology and the four systems used for generating NILV designs. Mutations or modifications in LVs and their safety are addressed with reference to pre-clinical studies. The detailed application of NILVs in promising pre-clinical studies is also discussed.

## 1. Introduction

Lentiviruses are a genus of retroviruses that include human immunodeficiency virus (HIV), simian immunodeficiency virus (SIV), bovine immunodeficiency virus (BIV), feline immunodeficiency virus (FIV), puma lentiviruses, and equine infectious anaemia virus (EIAV). Among these viruses, HIV is widely used and has become the standard for lentiviral vectors (LVs) to facilitate the delivery of genetic material (DNA or RNA) into target cells [1,2]. HIV-1 derived LV is well known for its efficient and stable transduction in dividing and non-dividing cells. It integrates the desired transgene into the target cell genome using a viral integrase enzyme. In addition, LVs have the advantage of broad cell tropism and target-specific cell types through pseudotypes.

LVs can be stably integrated into the target cell genomes as proviruses. The three generations of LV-packaging systems are safer and are suited for the production with HEK293T cell line, while several studies reported increased vector production by utilising SV40 T-antigen [3,4]. Vectors yielded at 48 and 72 h post-transfection can be harvested from the supernatant. Further purification and concentration protocols should be performed to produce debris-free LVs. Due to the integration of provirus at the random site of a target genome, LVs may induce potential DNA mutations and detrimental side effects such as tumour formation [5,6]. LV-infected cells can become cancerous either by oncogene activation or tumour suppressor gene inactivation. It is also possible for insertional mutagenesis to occur due to gene dysregulation at the LV integration site within or near a coding region of the host genome [6]. In 2005, one study reported a high incidence of lentiviral-vector-associated tumorigenesis following in utero and neonatal gene transfer in mice [7]. Decreasing the efficiency of the integrase enzyme by inducing mutations in IN enzyme will impair the provirus formation, although vector DNA will exist as non-replicating episomes in the transduced cells. Thus, dividing cells gradually lose the episomal DNA, while the insertional mutagenesis is reduced. Alternatively, non-integrating LV (NILVs) can be designed by introducing mutations in the LV integrase or Δatt (LTR integrase attachment) site [8,9]. NILVs were optimised and modified to increase the transgene expression and to improvise the safety profile [10]. NILVs were developed to aid in gene transfer and deliver advancements in clinical applications, especially in cellular reprogramming and genetic therapy. Transient gene expression in NILVs is the ideal choice for cytotoxic-cancer therapies, protective NILV immunisation and episomal expression [11]. In this review, we first describe the biology of different LVs, the design of NILVs, and their limitations with respective to the possible solutions, which will be described. Next, the clinical applications of NILVs in different areas such as vaccination, cell-type differentiation, site-directed integration, and gene therapy will be reviewed.

## 2. Lentiviral Vector Biology

**Figure 1:**
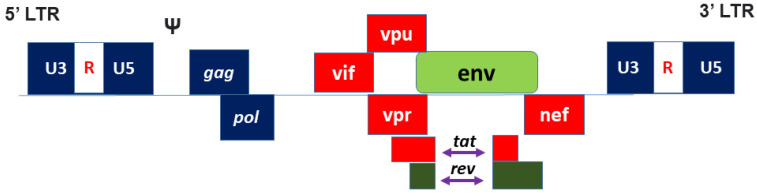
The HIV-1 virus contains three gene regions, *gag*, *pol*, and *env*, along with accessory genes *(vif*, *vpr*, *vpu*, *nef*), regulatory genes (*tat*, *rev*), and the 5′ and 3′ flanking long terminal repeats (LTR). The psi (Ψ) element is located at the 5′ end of the HIV-1 genome just upstream of the *gag* initiation codon.

**Figure 2:**
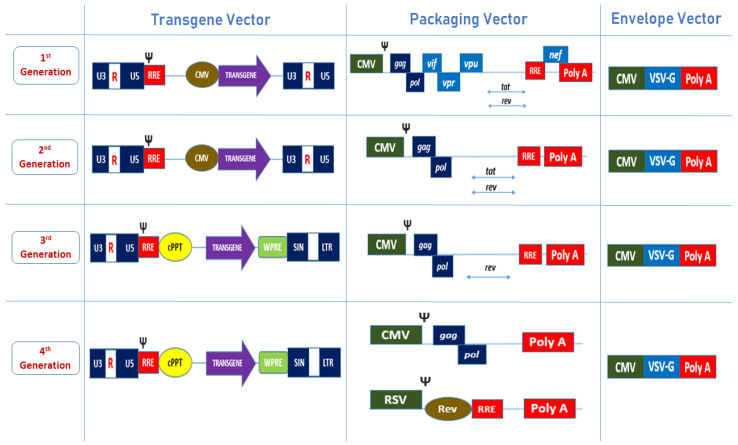
The basic four-generation lentiviral vector plasmid system. The transgene vector is similar for both first- and second-generation lentiviral vectors, while the third-generation lentiviral vectors additionally have a central polypurine tract (cPPT) and woodchuck hepatis virus post-transcriptional response element (WPRE). SIN vectors also replace the U3 region of 3-LTR in both third- and fourth-generation LVs. The packaging vectors differ for the four generations. The first-generation lentiviral vector has all of the accessory genes required for viral replication (*vif*, *vpr*, *vpu*, and *nef*). In contrast, second-generation lentiviral vectors do not have these accessory proteins in their packaging vector. Third-generation LVs do not have the Tat regulatory protein. The fourth generation is differentiated by the split of the *gag*/*pol* and *rev* sequences into two different cassettes. All four systems contain VSV-G as an envelope vector.

Lentiviruses reverse transcribe their genomes from RNA into DNA after entering the target cells. The HIV-1 genome is constituted by essential viral elements that are flanked between non-coding sequences that control gene transcription and protein synthesis, as shown in Figure 1 and Table 1 There have been many clinical trials involving lentiviral vectors and a product has already been approved in Europe (Zynteglo). However, this product has been undergoing investigation due to safety concerns since February 2021 [12]. Therefore, more studies on LV safety profiles should be given priority, as they are derived from the pathogen HIV-1. The removal of certain accessory proteins and the splitting of the genomes into multiple plasmids were first performed to decrease the formation of replication-competent lentiviral vectors (RCLs) and to reduce adverse consequences. These modifications were then divided into three major generations of vectors, as shown in Figure 2. The third-generation vector system consists of 4 plasmids introduced into cells via transient transduction. Self-inactivating (SIN) plasmids in the third-generation system harbour a large deletion of TATA box, Sp1, and NF-kB transcription factor binding sites that further reduce viral replication [13]. SIN vectors are now largely used in upcoming and ongoing clinical trials, as listed in Table 2. The recent fourth generation of LVs involves a five-plasmid system used to further minimise the probability of recombination events that might generate viruses that are capable of autonomous replication [14]. In the fourth-generation systems, *gag* and *pol* sequences are located in the same transcriptional unit, while rev is on a separate plasmid. This reduces the possibility of creating RCL events compared to the third-generation system, and in fact this generation is considered the safest to date [15]. However, few studies on third-generation LV systems have reported on RCL events as well [16,17].

## 3. Non-Integrating Lentiviral Vector (NILV) Design

A vital component of the integration complex of LV is the viral integrase enzyme (IN) that catalyses viral DNA integration into the host genome [18,19]. IN mediates the integration between vector and host DNA as the integration complex reaches the nucleus. However, this process carries predictable risks of harmful insertional mutagenesis, prompting an examination of alternatives to vector-mediated integration. To address this, NILVs were established. NILVs can stably express transgenes from the episomal DNA in non-dividing cells or transiently if the target cells divide both in vitro and in vivo. This also prevents the provirus formation while the vector DNA remains one of the primary episome types [10]. Few approaches were revised in this generation of NILVs. NILVs were developed by mutating the integrase gene or by modifying the attachment sequences of the LTRs, and these mutations were divided into class I and II mutations according to their effects, as shown in Table 3.

Class II mutations can be performed by exchanging the standard *gag* or *pol* packaging plasmid with an IN mutant strain [25]. However, several studies reported that mutations in specific amino acids of IN resulted in impaired integration of lentiviruses [8,26]. As class II mutations are mostly involved in altering the three protein domains in IN, namely the N-terminal domain, C-terminal domain, and IN core domain [27], they can affect the reverse transcription as well. Mutations introduced into IN not only affect the integration but also other relevant processes, especially by impairing the vital viral life cycle stages and causing pleiotropic effects that can make them unsuitable for vector development.

In contrast, class I mutations are limited in affecting the integrase multimerisation, linear episome processing, and DNA binding [25], as listed in Table 3. Mutations at a specific region will specifically target the integrase activity while keeping the other viral stages intact. The IN core domain has D64, D116, and E152, which are collectively known as a catalytic triad [28]. Any alterations to these amino acids will inactivate the catalytic activity of IN and inhibit the integration while preserving the transgene expression [20,29,30]. Additionally, mutations at W235, N120, and RRK (262–264) specifically block the genomic DNA binding. Alterations of H12 affect the IN multimerisation to generate NILVs. In addition, K264/K266/K273 mutations, also known as triad mutations, have been proven to impair both the DNA binding and strand transfer. Mutated 3′ LTRs CA/TG dinucleotide gave rise to LTR att mutants and also make successful NILVs remain functional and efficient [30].

### 3.1. Comparisons of NILVs with Other Non-Integrating Methods

Together with NILVs, various methods and options were also identified and studied for their efficiency in pre-clinical studies, as the elimination of transgene integrations is one of the major goals. Several integration-free approaches and their limitations are listed in Table 4, all of which have their own limitations. Among these methods, episomal plasmid and Sendai viral (SeV) vector are the methods that have highest efficiency and with only occasional integration. Therefore, the NILV approach was compared to episomal plasmid and SeV methods to examine its advantages and limitations before proceeding with NILVs as a better option in clinical applications.

SeV vector, an RNA viral vector, is one of the most valuable tools in human iPSC generation. It is also well-known for its high transduction efficiency compared to other methods [63]. Another recent study showed the ability of SeV vector to produce small regulatory RNAs with high transduction efficiency, durable expression, low cytotoxicity, and less risk of chromosomal insertion [32]. Although the SeV vector is known as being “ex-gene-free”, several researchers are still unsure of its safety in producing clinical-grade iPSC lines, as the avoidance of transgene integration passively depends on the cell passage [64]. Adding to this, the expression levels of the genes may vary according to the infected cells, and this might affect the quality of the generated iPSC lines. Therefore, the long-term stability of the genome structure and epigenetic conditions must be thoroughly examined.

Although the SeV vector is considered safe due to its integration-free ability, these kinds of virus-based vectors have a size limitation in terms of their carriages compared to plasmid-based vectors, which theoretically have no restrictions in the size of their carriages. Thus, as an alternative option, researchers also studied Epstein–Barr virus (EBV) episomal plasmids, which have stable gene expression and long-term plasmid retention, especially in dividing cells [65]. In addition, EBV has a cargo packaging capacity that overwhelms this vector compared to adeno-associated virus (AAV). However, EBV is not superior to the AAV vector because this virus vector does not integrate into the host genome and is non-pathogenic. AAV also has been shown to have limitations in a few gene therapy studies, such as its toxicity and unnecessary immunoreaction, especially in neuroscience research [66,67]. Recently, one research group used a combined gene delivery method in which the EBV nuclear antigen (EBNA) and its OriP fragment with recombination sites were incorporated into the baculoviral vector. This fragment was then recombined in a host cell to create an episomal vector. This approach had high transgenic efficiency [68].

NILVs exhibit higher transfer efficiency in not only in the dividing but also the non-dividing cells, attracting a wide range of researchers. Unlike LVs, NILVs do not present a risk of insertional mutagenesis. Moreover, NILVs are capable of transducing a broad range of cells or tissues with cargo packaging capacity compared to other vector systems. However, NILVs cannot introduce stable exogenous gene expression to the dividing cells, thereby limiting their application in clinical settings. Thus, adding modifications to the NILV design is one of the popular options. For example, Xu et al. added a minimal scaffold–matrix attachment region (S/MAR) sequence (SNIL) and successfully proved that this method manages to retain the episomal transgene expression in the dividing cells [69].

### 3.2. Limitations of NILVs and Possible Solutions

As mentioned in the previous section, NILV has drawbacks that need to be corrected. Some major drawbacks can be fixed through simple modifications or by choosing NILVs wisely for appropriate studies. For example, NILV-transduced cells will harbour the circular non-replicating nuclear episomes but without the capability of replication, resulting in the loss of the NILV episomes during rapid division and limiting their use for long-term expression. Therefore, NILVs can be a great option for less-proliferating or slower-dividing cells such as mesenchymal stem cells (MSCs), but are not suitable for highly proliferating cancer cells.

Furthermore, NILVs also confer reduced transgene expression compared to their integrating versions. This can be amended by using strong enhancers or promoters that can produce sufficient transgene expression [70,71,72]. On the other hand, one can also reduce the use of inhibitors to episomal transgene expression, by removing *cis*-acting genes, although this is still insignificant [73] and requires further studies. Another approach to enhance the expression of integrase-defective LVs is by retaining the Vpr accessory protein via transcriptional activation of the HIV-1 LTR [74]. However, studies found that Vpr induces global remodelling of the cellular proteome and could be responsible for the silencing of the unintegrated virus. Thus, one study suggested that Vpr itself can antagonise this silencing by degrading the SMC5-SMC6 complex localisation factor 2 (SLF2), which is responsible for the restriction of gene expression in the unintegrated viral genomes.

On the other hand, the residual integration of NILVs is still not accepted for certain clinical applications, although these integrating pro-viruses are non-canonical and would not necessarily display all of the characteristics of wild-type proviruses. Thus, NILVs are still the best option for quiescent cells, as critically summarised in one review paper [75]. High transgene expression with reduced integration while maintaining low-level vector episomes should be the ideal goal for successful NILV development. Since the integrative lentivectors are useful for some applications, especially for achieving long-lasting persistence, avoiding the presence of integrated proviruses in the cells would strongly reduce the therapeutic effect. However, we cannot deny that the vector persistence in every transduced stem cell can potentially influence the safety profile in the clinical studies of the diseases.

## 4. Clinical Application of NILVs

LVs are among the most efficient gene transfer tools for dividing and non-dividing cells. However, insertional mutagenesis has been found in clinical trials with lentiviral vectors, prompting a detailed study of genotoxicity assays of all integrated vectors [76,77]. Avoiding integration is the most direct approach to overcome this problem for many clinical applications. This can be facilitated through extensive studies of the integrating mechanisms of lentiviruses, as discussed earlier. NILVs have been studied for the treatment of infectious and genetic diseases, in addition to being great cell mediators for reprogramming. From our point of view, the transient expression of NILVs over stable expression is also highly preferable for vaccinations, cell type differentiation, site-directed integration, and persistent episomal expression. This is due to the progressive loss of the transgenes expressed from these non-integrated proviral forms.

### 4.1. Vaccination

Transient expression of NILVs is required for vaccine development, as it shows sustained immune responses against various diseases in pre-clinical models, as shown in Table 5. Apart from cellular and humoral immunity, NILVs have also been proven successful for sustaining anti-tumour immunity. Dendritic cells (DC) are the most common targets for vaccines. They represent a safe and efficacious vaccination platform for the development of prophylactic vaccines. Many studies used dendritic cells as targets for malaria, thymoma, hepatitis B virus (HBV), and many more infectious agents that can be treated with NILVs. In a pre-clinical study, a single immunisation of NILVs encoded in a secreted form of the envelope of a virulent strain of West Nile virus (WNV) induced a strong B cell response, and a single immunisation was also sufficient to induce early and long-lasting protective immunity [78,79]. On the other hand, one in vivo study involving the H5 influenza A virus (IAV) demonstrated that the monoclonal antibody (mAb) administered through NILVs did not persist for longer time points [80]. However, this could be studied further to make it an effective strategy for rapid protection against infectious diseases in the future. Vaccinia virus [81], Zika virus [82], and human cytomegalovirus [83] have also been investigated in pre-clinical studies to test their immune responses.

Additionally, the injection of the DC-directed IDLV encoding OVA has shown promising immune responses in C57BL/6 mice and complete protection against a thymoma tumour expressing a delivered ovalbumin (OVA) antigen in mice [87]. This shows that the IDLV system represents a promising and efficient vector platform for the future development of DC-based immunotherapy. Furthermore, cancer cell vaccines were also developed as an alternative approach to DC, since cancer cells express tumour antigens of interest and show robust improvements. Human immunodeficiency virus (HIV-1), human papillomavirus (HPV), and hepatitis C virus (HCV) were tested with cancer vaccines targeting the antigen-presenting cells [23]. Developing a strong cellular immunity among HIV patients has been the aim of several clinical studies. Therefore, several studies have suggested working on SIV-based NILVs to evaluate the induction of transgene-specific immune responses against sensibly designed structural HIV antigens, as proven in BALB/c mice [88,89]. This could be very efficient for studying the T cell immunogens for the development of long-lasting and effective HIV vaccines. A study showed that NILVs injected intramuscularly express systemic CD8+ T cells and antibody responses to the secreted hepatitis B virus (HBV) surface antigen and have also been proven as a great tumour therapy [90].

As a cancer immunotherapy candidate, NILVs delivering the tumour antigen NY-ESO-1 to the human dendritic cells in vivo have shown promising clinical results by showing sustained CD8+ T cells within 14 days post-immunisation and anti-tumour responses [91,92]. In 2020, this vaccine was improved with a heterologous boost by priming it with a recombinant protein, adenoviral vector, or self-replicating RNA boost. This resulted in increased efficacy of ZVex as a cancer vaccine. Improving the existing vaccines is a better option compared to developing new vaccines [86].

Another exciting study involved the application of NILVs to generate a vaccine candidate against coronavirus disease 2019 (COVID-19). Golden hamsters, which are naturally tolerant to SARS-CoV-2 replication and closely mimic human COVID-19 physiopathology, showed significant vaccination effectiveness and could limit lung deleterious damage by utilising the NILV approach [85]. These findings showed that NILV-based intranasal vaccination against SARS-CoV-2 has a significant prophylactic effect and is a viable option against COVID-19.

### 4.2. Cell-Type Differentiation

Regenerative medicine involving pluripotent stem cells has become an ideal goal for many researchers. Cellular reprogramming is another great application of NILVs via transient expression, as shown in Table 6. Additionally, iPSC production and differentiation into the lineage of interest is an alternative approach to the use of embryonic stem cells (ESCs). Furthermore, iPSCs can be an outstanding alternative to embryonic stem cells, although have several limitations and ethical concerns. Additionally, iPSCs also have similar pluripotency ability to human ESCs. As such, iPSC technology could be the desired application method for NILVs, as fewer genomic abnormalities are expected.

Successful transformation of human somatic cells into iPSCs was first carried out in 2007 using a lentiviral system [95]. Since then, multiple approaches were taken by other researchers worldwide in generating iPSCs with additional transcription factors, such as Oct4, Sox2, KLF4, and C-myc (OSKM) [96], while concentrating on higher safety profiles in clinical settings. Certain transcription factors are known as oncogenes and possible side effects are foreseeable [97]. Nonetheless, reprogramming without viral integration with plasmids or direct reprogramming protein delivery assays can solve this issue [98,99]. Additionally, several more recent technologies and approaches have been suggested for producing iPSCs without transgene integration that can cause possible mutagenesis, such as non-integrating viral vectors, RNA virions, RNA replicons, non-integrating replicating episomal plasmids, minicircles, Cre-loxP excision of transgenes, excisable transposon, protein transduction, RNA transfection, microRNA transfection, polycistron, and pre-integration of inducible reprogramming factors [42,100]. Polycistronic technology reduces the requirement for multiple viral vectors to deliver four different transcription factors, cutting this down to a single-stem cell cassette [101]. A study by Awe et al. compared the reprogramming efficiency of the polycistronic stem cell cassette (STEMCCA) to other integration-free approaches [102]. A similar polycistron approach was slightly modified using the Cre-Lox method. Another study was performed [103], whereby the integrated transgenes were excised from the reprogrammed genomes using the Cre-Lox method first and a single plasmid containing the four reprogramming factors was linked by the 2A sequence.

This type of advanced technology involving minimising genomic integrations can be used for potential human clinical trials with safer profiles. More studies are working on enhancing the iPSC reprogramming efficiency, such as by adding SV40 large T antigen (T) by 23–70-fold from human fibroblasts [104]. A recent study conducted a DNA methylation landscape assessment for isogenic iPSCs to compare different reprogramming methods, which could also be a way of proving the safety of iPSC lines generated by reading any significant changes in DNA methylation profiles [105].

Various platforms can be used to generate iPSCs through non-integrating methods, such as by transient transfection, EBV episomal plasmids, synthetic mRNA, adenoviral vectors, microRNA mimics, minicircles, and SeV vectors. However, all of these methods have low re-programming efficiency, except the Sendai viral vector [100]. NILV-based iPSCs can be used to study stem cell biology, as a cellular platform for pharmacological and toxicological studies, and can be considered as a possible source of autologous stem cells for use in regenerative medicine. Additionally, iPSCs have a great advantage in replacing human tissues or cells for disease modelling, drug screening (toxicity and efficacy), and cell-based therapy, especially for cardiovascular diseases [106].

However, most studies have used other non-integrated approaches, as discussed earlier, and only a few studies have used the NILV approach to produce iPSCs. In a previous study, purified integrase-deficient LV facilitated the generation of a population of purified hESC-derived hepatic progenitors that were devoid of integrated viral DNA. The ESCs could be differentiated into specific progenitor cells. For example, human embryonic stem cells (hESCs) encoding for green fluorescent protein (GFP) driven by the liver-specific apolipoprotein A-II (APOA-II) promoter can be differentiated into hepatic progenitors at day 16. Surprisingly, 99% of these APOA-II-GFP-positive cells expressed the hepatoblast markers, such as α-fetoprotein and cytokeratin-19, and were further cultured into more mature cells that resembled the hepatocytes [93]. This approach can be used in cell therapy and in in vitro applications, such as drug screening. The reprogramming efficiency of NILV has not been well-explored or compared with the SeV approach in vitro. Therefore, evidence is needed in the future of the dynamic effects on genomic stability.

To the best of our knowledge, iPSCs in clinical trials mostly involve neurological and cardiovascular disease treatments (such as for Parkinson’s disease) via iPSC-derived HSCs and cardiac cell injury via iPSC-derived cardiomyocytes. Japan started the world’s first iPSC-based clinical study in 2013, whereby a macular degeneration patient received a transplant of iPSC-based retinal cells; however, the patient showed no improvement [107]. Another study at Kyoto University focused on dopaminergic precursor cells that were differentiated from iPSCs injected into the brain of a patient with Parkinson’s disease. This boosted the dopamine levels and improved the patient’s symptoms [108,109].

### 4.3. Site-Directed Integration

NILV is also used as a template for site-directed integration systems. A variety of systems are available for direct integration into genomic “safe sites” or for gene-specific correction to minimise the dysregulation of gene expression, as listed in Table 7. One approach involves combining a recombinase or transposase transiently with an NILV to facilitate integration at specific sites. The most recent example involved the use of the sleeping beauty (SB) transposon and transposase expression cassette. NILVs were able to facilitate transient transposase expression to the target cells. Several studies using NILVs have shown similar integration to the SB cassette without viral element integration, thereby reducing the insertional mutagenesis [110]. An HIV-1/SB hybrid vector facilitated by the hyperactive SB100X transposase that allows efficient DNA transposition in primary human cells could be a valuable tool for therapeutic gene transfer, as it could be inserted into actively transcribed genomic regions. Another study explained that non-viral yeast Flpx9 recombinase produced by NILV can enter the circular viral recombination substrates and facilitate the site-directed genomic insertion [111].

Moreover, NILVs can be designed to promote site-specific homologous recombination (HR). This includes NILVs combined with a rare cutting nuclease for targeted recombination at specific sites by HR. This will lead to successful targeted gene correction. For example, an NILV encoding a repair template was co-transported with an I-SceI nuclease expression vector to rescue a defective enhanced green fluorescent protein (EGFP) gene. Expression of the nuclease created a double-strand break (DSB) within the targeted locus and stimulated stable gene correction at the end of the process [21]. Adding to this, calmegin (clgn) targets the neomycin-resistant transgene cassette that generates ES cells via transgene insertion. However, this method is unbeatable against the traditional electroporation method, which allows for more efficient gene transfer [116]. Thus, better modifications and changes are the main concerns of many researchers. NILVs can also be used as templates for HR along with engineered zinc finger nucleases (ZFNs) [117,118] and transcription activator-like effector nucleases (TALENs) [115] to induce successful gene correction at the target gene locus with less off-target integration. Recently, the development of regularly clustered, interspaced, short palindromic repeat and CRISPR-associated protein 9 (CRISPR/Cas9) technology has made NILVs ideal tools. CRISPR/Cas9 and a guide RNA targeting the cytochrome b-245 heavy chain can correct the defects in hematopoietic cells [119]. Another study also utilised CRISPR/Cas9-mediated, homology-directed repair via an ex vivo approach to conduct gene correction for recessive dystrophic epidermolysis bullosa [120].

### 4.4. Gene Therapy

From our viewpoint, the use of NILVs in gene transfer or gene therapy is a promising strategy for delivering therapeutic genes for genetic diseases such as cancer, macular degenerations, heart disease, diabetes, haemophilia, AIDS, and most blood-related disorders that is equal to the use of LVs. The LV-based gene therapies known as Zynteglo [121] and Libmedly [122] have been announced as treatments for β-thalassemia and metachromatic leukodystrophy (MLD), respectively, by the European Medicines Agency (EMA). However, this may to indicate a certain level of uncertainty related to future products, which may further delay the success and approval of LV-based therapies. Therefore, NILVs as alternatives with proven success in gene therapy can also be approved to meet the ultimate goal of treating genetic disorders without detrimental risks. Researchers have been replacing mutated and defective genes and making diseased cells more evident to the immune system. Gene transfer is a challenging process, as poorly studied unsuccessful steps can lead to adverse immune system reactions, incorrect cell targeting, infections caused by the virus, and tumour growth. For example, SCID-X1 patients who received HSCs transduced with murine leukaemia virus (MLV) developed abnormal T cell proliferation due to the insertion of a retroviral vector near the LMO2 proto-oncogene [123,124]. In another study, a successful dopamine replacement gene therapy in patients with Parkinson’s disease performed by targeting important genes for dopamine synthesis regulated the dopamine concentration, proving that gene therapy can be successful [125]. Therefore, gene therapy involving NILVs also needs to be assessed in order to bring this method into clinical application, since it has been proven to be better and more successful than other gene delivery methods, as listed in Table 8.

NILVs encoding for in vivo expression of GFP have been reported in organs such as the brain, liver, and spinal cord via stable gene transfer for longer periods (up to months) in several studies [22,73,131,132,133,134]. In another study, hepatocytes that target NILVs with coagulation factor IX (FIX) in haemophilic mice induced an immune tolerance [135]. A similar study using FIX transgenes carrying an R338L amino acid substitution associated with clotting hyperactivity and thrombophilia showed increased gene therapy efficacy but also less efficiency in hepatic transgene expression of NILVs [136]. Recently, another study demonstrated that the NILV approach can overcome immune rejection and allow for the growth of transduced cells in an immunocompetent host by producing CRISPR-modified murine cell lines using mutated integrase vectors [137]. In addition to the advantages of the CRISPR/Cas9 system, another study proved that a Cas9 protein delivery system with NILVs encoding both guide RNA and donor DNA resulted in efficient DNA breakage, one-time genome correction of the sickle cell disease (SCD) mutation, and long-term engraftment of HSCs [126]. These studies explained the use of combined NILV approaches for a safer, long-lasting, and fruitful outcome in future research. Gene therapy has been given more importance compared to other clinical applications in treating many genetic disorders that are highly disruptive to human health. The latest research has also demonstrated a successful combination gene therapy strategy for HIV using a two-vector system design, which uses an integrating LV to transduce the cells sequentially and a non-integrating lentiviral LV to insert the conditional suicide gene, with knockout of CCR5, and transient expression of GFP to enrich the modified cells [128].

## 5. Future Directions and Challenges

From the frame of reference of this review, the integration-free capability of NILV is highly desirable for gene therapy and could be the gold option for vaccination and cell immunisations studies with long-lasting immune responses. However, very limited or less studies have chosen NILVs as a gene delivery method to generate iPSCs, although this approach has potential. The earlier iPSC reprogramming work utilised the viral vector system to express the advantages of OKSM with a highly successful rate. This opened up new possibilities in regenerative medicine, especially for the development of disease-specific models, including in drug toxicity studies. However, this viral vector also causes multiple potentially harmful integrations of the transgenes into the host genome, which lead to tumour formation. Although non-viral methods such as protein transduction [138], MiRNA expression [60], and mini circle vector expression [48] have been introduced to eliminate this harmful effect, the efficiency of generating functional iPSCs using the viral system means it is still favoured. Therefore, the effort to refine and develop alternative approaches is being continued, including the use of non-integrating systems to deliver the reprogramming genes.

In addition, a study published a decade ago showed for the first time the successful reprogramming of blood cells using non-integrative SeV as an efficient integration-free gene delivery method [139]. The high reprogramming efficiency without genomic integration of the SeV vector was also demonstrated commercially [140]. Since iPSCs have not been generated using the NILV approach, the same concept can be expected to be applied using NILVs with a similar safety profile and with a slight possibility of transgene integration into the host in the future. This increases the hope of working more on NILVs by setting SeV vectors as an ideal example to generate successful high-clinical-grade iPSCs. In conclusion, research is still ongoing at every step of therapeutic application for NILVs in the hope of achieving better curative options in the future.

The major drawback of NILVs are their reduced transgene expression and gradual loss of episomal vectors, especially in dividing cells, which eventually reduce the therapeutic effect and mean it does not have long-lasting persistence. Some important improvements that can be further explored in NILV systems are the introduction of multiple mutation sites of the integrase sequence gene. This approach could help to increase the episomal gene expression, as reported in a previous study where different mutation sites of the integrase plasmid resulted in different gene expression levels [75]. Another suggestion is to use a different type of promoter to drive the expression of the pluripotent genes, as the current promoter, the CMV promoter, is likely to be highly methylated during prolonged expression and might not be suitable or efficient for episomal expression due to the high replication rate of the transduced cells [141,142]. Only targeted methylation-induced gene silencing could be reversed via the addition of 5-aza-2′-deoxycytidine. Alternatively, another option is to consider introducing a transduction domain at the 3′-5′ exonuclease of the transcription gene, which could boost and enhance the gene expression, thereby increasing the reprogramming efficiency using NILVs.

Additionally, as NILVs have been used as the best option for non-invasive gene-based imaging only in non-dividing cells [143], highly proliferating cells should be considered for use with this approach in the future by merging OriP plasmid technology from EBV into NILV episomes that can retain the plasmids. Apart from the SNIL vector, incorporating the simian virus 40 (SV40) into NILVs could maintain the episomal DNA in dividing cells. One concern in merging or modifying the original vector to fix these limitations is the abnormal mutations and infections, which could multiply the existing limitations. Thus, careful epigenetic studies and DNA methylation profiles are needed prior to successfully applying the NILV approach in clinical settings.

In a nutshell, NILVs have emerged as an important tool in biomedical research as a therapeutic option. Future studies are needed with more advanced human clinical trials to achieve improvements and for optimisation in order to increase the safety and reduce illegitimate integration. The application of NILVs in the pre-clinical trials of recombinant protein production, vaccines, gene therapy, cell imaging, and induced pluripotent stem cell (iPSC) generation should be explored further to bring about even more advancements in the future.

## Figures and Tables

**Table 1:** Functions of HIV-1 proteins.

	Gene	Size of Proteins	Protein	Function
**Structural Proteins Proteins**	*gag*		Pr55Gag	precursor of the inner structural proteins
		p24	capsid protein (CA)	forms the conical capsid
		p17	matrix protein (MA)	forms the inner membrane layer
		p7	nucleoprotein (NC)	forms the nucleoprotein–RNA complex
		P6	52aa precursor of Pr55Gag	involved in virus particle release
	*pol*		Pr160GagPol	precursor of the viral enzymes
		p10	protease (PR)	release of structural proteins and viral enzymes
		p51	reverse transcriptase (RT)	transcription of HIV RNA in proviral DNA
		p15 (66)	RNase H	degrade viral RNA
		p32	integrase (IN)	integrates proviral DNA into the host genome
	*env*		PrGp160	precursor of the envelope proteins SU and TM
		gp120	surface glycoprotein (SU)	attaches the virus to the target cell
		gp41	transmembrane protein (TM)	fusion of viral and cell membranes
**Regulatory Proteins**	*Tat*	p14	transactivator protein	activates the transcription of viral genes
	*rev*	p19	RNA splicing regulator	regulates the export of non-spliced and partially spliced viral mRNA
	*Tev*	p26	tat/rev protein	Tat-Env-Rev fusion protein regulates the activity of Tat and Rev in the nucleus
**Accessory Proteins**	*nef*	p27	negative regulating factor	affects HIV replication, enhancement of infectivity of viral particles
	*vif*	p23	viral infectivity protein	infectious virus production
	*vpr*	p15	virus protein r	facilitates virus infectivity, affects the cell cycle
	*vpu*	p16	virus protein unique	virus particle release, regulates intracellular trafficking.
	*vpx*	p15	virus protein x	involved in early steps of virus replication of HIV-2

**Table 2:** The clinical trials conducted using self-inactivating lentiviral vectors.

Started Year	Study Title	Diseases/Conditions	Interventions	Citations
2017	Gene Transfer for SCID-X1 Using a Self-Inactivating Lentiviral Vector (TYF-IL-2Rg)	Severe Combined Immunodeficiency (SCID), X-Linked	Biological: TYF-IL-2Rg gene-modified autologous stem cells	https://clinicaltrials.gov/ct2/show/NCT03217617 (accessed on 25 May 2021)
2017	FANCA Gene Transfer for Fanconi Anaemia Using a High-Safety, High-Efficiency, Self-Inactivating Lentiviral Vector.	Fanconi Anaemia	Genetic: Gene-modified autologous stem cells	https://clinicaltrials.gov/ct2/show/NCT03351868 (accessed on 25 May 2021)
2018	Gene Transfer for ADA-SCID Using an Improved Lentiviral Vector (TYF-ADA)	Adenosine Deaminase Severe Combined Immunodeficiency (ADA-SCID)	Genetic: TYF-ADA gene-modified autologous stem cells	https://clinicaltrials.gov/ct2/show/NCT03645460 (accessed on 25 May 2021)
2018	Lentiviral Gene Therapy for MLD	Metachromatic Leukodystrophy (MLD)	Biological: Lentivirus-mediated delivery of ARSA to the CNS	https://clinicaltrials.gov/ct2/show/NCT03725670 (accessed on 25 May 2021)
2018	Autologous Gene Therapy for Artemis-Deficient SCID	Severe Combined Immunodeficiency (SCID)	Drug: AProArt Device: CliniMACS^®^ CD34 Reagent System cell sorter device Drug: Busulfan	https://clinicaltrials.gov/ct2/show/NCT03538899 (accessed on 25 May 2021)
2019	Gene Therapy with Modified Autologous Hematopoietic Stem Cells for Patients with Mucopolysaccharidosis Type IIIA	Mucopolysaccharidosis Type IIIA	Drug: Autologous CD34+ cells transduced with a lentiviral vector containing the human SGSH gene	https://clinicaltrials.gov/ct2/show/NCT04201405 (accessed on 25 May 2021)

**Table 3:** Summary of point mutations and affected functions in IN that have been used to develop NILVs.

Mutation Type	Sites of NILV Mutation	Steps Affected	References
H12	N-terminal domain	IN multimerization	[20]
D64	Core domain	Inactivates catalytic properties of IN	[21,22]
D116	Core domain	Inactivates catalytic properties of IN	[21,23,24]
N120	Core domain	Impairs binding to genomic DNA	[20]
Q148	Core domain	Vector DNA binding	[20]
E152	Core domain	Inactivates catalytic properties of IN	[21]
W235	C-terminal domain	Impairs binding to genomic DNA	[20]

**Table 4:** The advantages and disadvantages of integration-free methods.

Integration-Free Methods	Advantages	Disadvantages	References
SeV vector	- High transduction efficiency - Broad tropism - Low cytotoxicity	- Immunogenic - Multiple vectors required - Screening for integrations	[31,32,33,34]
Transient transfection	- No transgene integration	- Low efficiency - Multiple transfection - Vector silencing	[35,36,37]
EBV episomal plasmids	- Low immunogenicity - Single transfection - Reasonable efficiency - Cargo capacity - Long-term plasmid retention	- Screening for integrations	[36,38,39,40,41,42,43]
AAV vector	- No transgene integration	- Less capacity - Less efficiency - Transient expression	[44,45,46,47]
Minicircle	- Low immunogenicity	- Less efficiency - Multiple transfection - Occasional integration	[48,49,50,51,52,53]
Synthetic mRNA	- No transgene integration	- Less efficiency - Innate immune response - Multiple transfections - Challenging transfection	[54,55,56,57]
MicroRNA mimic	- No transgene integration	- Less efficiency - Multiple transfection - Transient expression	[58,59,60,61,62]

**Table 5:** Summary of elements used in developing non-integrating lentiviral vectors (NILVs) for vaccinations in pre-clinical studies conducted by the cited research teams. The first column lists the integrase mutations with subsequent columns categorizing the related diseases, target cells, and transgenes chosen for the mutations to be applied.

NILV Mutations	Disease	Transgene/Effector	Target	References
D64V	Influenza Virus	Influenza virus hemagglutinin (HA) and nucleoprotein (NP) transgenes.	Antigen-presenting cells	[84]
D64V	Zika Virus	ZIKA Protein codon-optimized	Dendritic cells	[82]
D64V	SARS-CoV-2	Codon-optimized nucleotide fragments encoding a stabilized, foldon-trimerized version of the SARS-CoV-2	Dendritic cells	[85]
D64V	CTA New York Esophageal Squamous Cell Carcinoma-1 (NY-ESO-1)	NY-ESO-1 gene	Dendritic cells	[86]

**Table 6:** Summary of elements used in developing non-integrating lentiviral vectors (NILVs) for cell-type differentiation in pre-clinical studies conducted by the cited research teams. Important components of vector systems are provided in the table.

NILV Mutations	Disease	Transgene/Effector	Target	References
D64V	Purification of hESC-derived progenitors	Green Fluorescence Protein	Hepatic Progenitor	[93]
D64V	iPSC transgene excision	Cre recombinase	iPSCs	[94]

**Table 7:** A summary of the elements used in developing non-integrating lentiviral vectors (NILVs) for site-directed integration in pre-clinical studies. Different approaches for direct integration or site-specific modifications of safe genomic loci in different studies are listed with attached references.

NILV Modification	Disease/Application	Transgene/Effector	Target	References
D64V	β-thalassemia/site-directed integration and KLF1 gene modification	Zinc finger nuclease/ZFN donor template–GFP expression cassette	γ-globin and HbF expression	[112]
D64V	β-thalassemia/site-specific gene modification	Zinc finger nuclease/ZFN donor template	*SOX6* region	[113]
D64V	Fanconi anemia/gene targeting using ZFN and IDLVs	OCT4, SOX2, c-MYC, and KLF4 flanked by loxP sequences.	AAVS1 safe harbor locus in fibroblasts	[114]
D64V	Site-specific gene modification	Transcription activator-like effector nucleases/TALEN donor template	*COL7A1* gene	[115]

**Table 8:** Summary of integrase mutations with added elements used in developing non-integrating lentiviral vectors (NILVs) for gene therapy in pre-clinical studies with specifically targeted organs.

NILV Modification	Disease/Application	Transgene/Effector	Target	References
D64V	Sickle cell disease	Efficient GFP-to-YFP gene conversion with Cas9 protein	engraftable HSCs	[126]
D64V	Stable gene transfer	Episomal erythropoietin (EPO) gene expression	human cord blood CD34+ cells	[127]
D64V	Stable gene transfer	CCR5gRNA-CRISPR/Cas9 cassette and HIV Tat protein, HIV Tat-dependent thymidine kinase mutant SR39 (TK-SR39) and GFP reporter gene	CD34+ stem cells	[128]
D64V	Stable gene transfer	Green fluorescence protein	hematopoietic stem and progenitor cells	[129]
D64V	Maculardegenerations/stable gene transfer	Green fluorescence protein, *E2F2* gene	Eye	[130]
D64V	In situ gene correction	Green fluorescence protein, RNA-guided Cas9 endonucleases (RGNs)	Hematopoietic cells	[119]

## Data Availability

No new data were created or analysed in this study. Data sharing does not apply to this article.
